# A cis-regulatory module upstream of *deltaC* regulated by Ntla and Tbx16 drives expression in the tailbud, presomitic mesoderm and somites

**DOI:** 10.1016/j.ydbio.2012.07.002

**Published:** 2012-11-01

**Authors:** Leila Jahangiri, Andrew C. Nelson, Fiona C. Wardle

**Affiliations:** Department of Physiology, Development and Neuroscience, Cambridge University, Downing Street, Cambridge, CB2 3DY, UK

**Keywords:** CRM, Ntla, Tbx16, T-box transcription factors, *deltaC*

## Abstract

Somites form by an iterative process from unsegmented, presomitic mesoderm (PSM). Notch pathway components, such as *deltaC* (*dlc*) have been shown to play a role in this process, while the T-box transcription factors Ntla and Tbx16 regulate somite formation upstream of this by controlling supply and movement of cells into the PSM during gastrulation and tailbud outgrowth. In this work, we report that Ntla and Tbx16 play a more explicit role in segmentation by directly regulating *dlc* expression. In addition we describe a cis-regulatory module (CRM) upstream of *dlc* that drives expression of a reporter in the tailbud, PSM and somites during somitogenesis. This CRM is bound by both Ntla and Tbx16 at a cluster of T-box binding sites, which are required in combination for activation of the CRM.

## Highlights

► Ntla and Tbx16 directly regulate *deltaC* expression. ► Ntla and Tbx16 bind at a cluster of T-box sites in a cis-regulatory module (CRM) upstream of *deltaC*. ► This CRM drives expression of a reporter in the tailbud, PSM and somites during somitogenesis. ► The T-box site cluster is required for activation of the *deltaC* CRM *in vivo*.

## Introduction

Somites are formed sequentially from the presomitic mesoderm (PSM) under the influence of a molecular oscillator, the segmentation clock (Cooke and Zeeman, 1976; Pourquie, 2003). As new somites form at the anterior of the PSM, cells enter the posterior PSM from a progenitor cell population in the tailbud. In zebrafish, anterior trunk somites originate from lateral mesodermal cells that ingress during gastrulation whilst more posterior somites form from progenitor cells in the tailbud, which themselves arose from cells of the ventral mesoderm during gastrulation (Kanki and Ho, 1997; Kimmel et al., 1990).

No tail a (Ntla) and Tbx16 (mutated in *spadetail*; *spt*) are T-domain transcription factors that together specify all trunk and tail mesodermal cell fates. Both are expressed in the mesoderm from early blastula stages, and by early gastrula stages have overlapping domains in the ventral and lateral margin where trunk and tail progenitors arise. During somitogenesis *ntla* expression is confined to the notochord and tailbud, while *tbx*16 is expressed in the tailbud, presomitic mesoderm and adaxial cells. Recent microarray analysis at the 12–13 somite stage has shown that *tbx*16 expression oscillates in the PSM (Krol et al., 2011). *ntla*^−/−^ mutant embryos lack notochord and posterior tail somites (Halpern et al., 1993; Schulte-Merker et al., 1994), while *spt*^−/−^ mutant embryos fail to make trunk somites although tail somites are specified normally (Griffin et al., 1998; Ho, 1992; Kimmel et al., 1989). Double mutants lack all somites as well as other trunk and tail mesoderm revealing redundant roles for these two factors in specifying mesoderm (Amacher et al., 2002; Goering et al., 2003). Both factors are also required for normal cell movements during gastrulation and tailbud outgrowth (Ho and Kane, 1990; Kimmel et al., 1989; Martin and Kimelman, 2008, 2010; Row et al., 2011). Ntla is required to establish and maintain progenitor cells in the tailbud through establishing a positive feedback loop with Wnt signaling (Martin and Kimelman, 2008), while Tbx16 is required for cells to complete the epithelial to mesenchymal transition as they enter the PSM from the tailbud (Row et al., 2011). Thus, somites do not form in these mutant embryos because progenitor cells are either not generated or not channeled properly into the PSM. However, during normal embryogenesis, Ntla and Tbx16 may play another role in somite formation, through regulating genes that are expressed in the PSM and somites and that are required for segmentation. Recently, for instance, Ntla and Tbx16 were shown to regulate *deltaD* expression through a regulatory module in the 2nd intron (Garnett et al., 2009).

Notch signaling is essential for segmentation of somites and components of the pathway are believed to comprise the molecular oscillator (Holley et al., 2000; Holley et al., 2002). In zebrafish, for instance, the Notch pathway ligands *deltaC* (*dlc*) and *deltaD* (*dld*) are required for segmentation of the paraxial mesoderm since mutants in *dlc* (*beamter*; *bea*) lack segmented somites beyond the first 2–6 and mutants in *dld* (*after eight*; *aei*) lack somites beyond the first 7–9 (Jiang et al., 2000; Julich et al., 2005; van Eeden et al., 1996; van Eeden et al., 1998). Recent studies have shown that *dlc* also plays an essential role in the synchronization and amplification of oscillations in the PSM between neighbouring cells, while *dld* drives oscillations in the PSM (Julich et al., 2005; Mara et al., 2007). The mesodermal expression of *dlc* is initiated at 50% epiboly in the ventro-lateral margin and later is observed in the tailbud, PSM and somites, with its expression in the posterior PSM being cyclic (Haddon et al., 1998; Julich et al., 2005; Smithers et al., 2000).

Here we show that both Ntla and Tbx16 directly regulate the expression of *dlc* and are able to bind a cis-regulatory module (CRM) upstream of the *dlc* transcription start site that drives expression in the tailbud, PSM and somites of the developing embryo. T-box sites in this region are necessary for reporter gene expression, suggesting that Ntla and Tbx16 directly regulate the expression of *dlc* during somitogenesis. These results implicate Ntla and Tbx16 in patterning of the somites through regulating a gene that is involved in segmentation.

## Materials and methods

### Wild type fish and mutant alleles

AB, *ntla*^*b*195/+^, *sp*t^*b*104/+^ and *ntla*^*b*195/+^; *spt*^*b*104/+^ fish were reared as described (Westerfield, 2000).

### Chromatin immunoprecipitation, qPCR, sequencing

For each antibody condition, 300 75% epiboly or 12ss embryos were collected for chromatin immunoprecipitation and qPCR was performed as described (Morley et al., 2009; von Hofsten et al., 2008; Wardle et al., 2006) using previously characterized anti-Ntla and anti-Tbx16 antibodies (Amacher et al., 2002; Garnett et al., 2009; Schulte-Merker et al., 1992). For ChIP-seq, two independent reactions were performed on 5000 75–85% epiboly embryos as described (Morley et al., 2009; von Hofsten et al., 2008; Wardle et al., 2006) but with a cleavable oligo used in the ligation-mediated PCR that was removed after amplification. Illumina paired-end libraries were prepared according to manufacturers instructions, size selected and sequenced on the Illumina GAIIx platform. All reads were converted to Sanger FASTQ format and mapped to the Zv9 version of the zebrafish genome using Bowtie (Langmead et al., 2009) in Galaxy (Blankenberg et al., 2010; Giardine et al., 2005; Goecks et al., 2010). Alignments were performed using the following criteria: -n2-e70-m2-k2-best. Peak calling was subsequently performed using MACS (Zhang et al., 2008) using default parameters except as follows: −−mfold5 -pvalue1e-4. Peaks were visualized using IGB (Nicol et al., 2009).

### Motif searching analysis

**Fig. 1:**
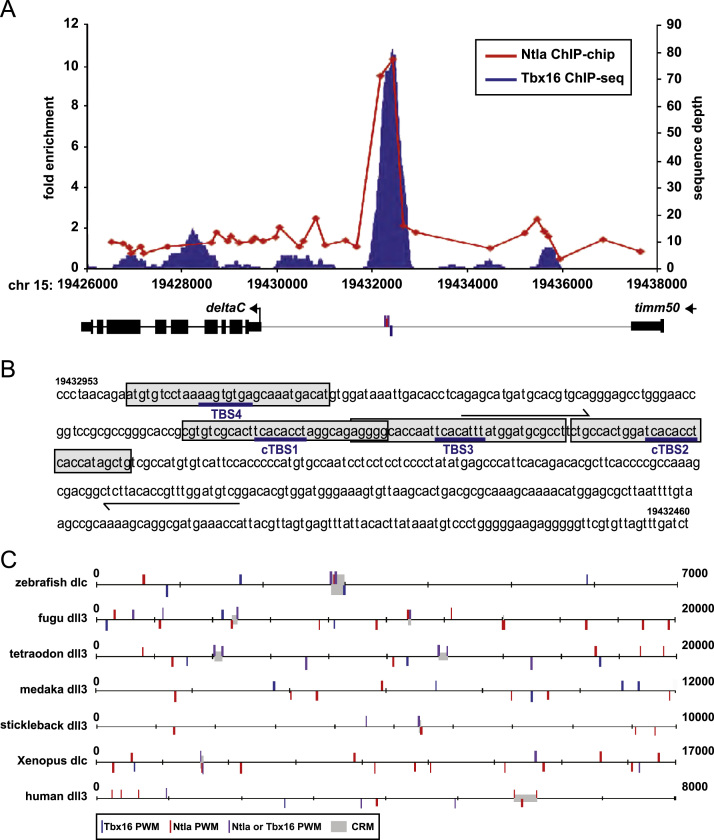
Ntla and Tbx16 bind to a CRM upstream of *dlc in vivo* (A) Red plot shows fold enrichment for Ntla ChIP-chip data. Blue plot shows sequence depth for Tbx16 ChIP-seq data. Chromosomal position (Zv9) is shown on the x-axis with the whole gene model for *dlc* shown below. The T-box binding site (TBS) cluster is indicated with coloured bars. (B) Sequence of the region under the Ntla/Tbx16 binding peak used in reporter gene assays, showing two consensus TBS (cTBS1 and 2) and two TBS with a 1–2 bp mismatch (TBS3 and TBS4). The grey boxes indicate the probes used in EMSA assays and the half arrows show the region amplified in ChIP-PCR (see Fig. S1). (C) Matrix-scan analysis of genomic region upstream of zebrafish *dlc* and tetraodon, medaka, fugu, stickleback, *Xenopus tropicalis* and human *dlc* orthologues. The interval from the *dlc*/*dll3* transcription start site (position 0) and the next upstream gene is shown. Blue bars indicate sites found using a Tbx16 position weight matrix (PWM), red bars indicate sites found using a Ntla PWM, purple bars indicate sites found using both the Tbx16 PWM and Ntla PWM, bars above and below the line indicate sites on the + and − strand, respectively. Grey boxes indicate CRMs predicted by the software (pval<0.0001).

Position weight matrices (PWMs) for Ntla, identified by SELEX and ChIP-chip (Garnett et al., 2009; Morley et al., 2009), and Tbx16, identified by SELEX (Garnett et al., 2009), were used to search genomic regions upstream of zebrafish *dlc* orthologues in fugu, medaka, tetraodon, stickleback, *Xenopus tropicalis* and human, and in introns in the case of medaka, using matrix-scan within the RSAT software suite (http://rsat.ulb.ac.be/). This software detects transcription factor binding sites and regions that are enriched in these sites (*i.e.*, CRMs) using pattern matching tools when given a PWM for a known transcription factor. A detailed description of the methods and statistics used to predict these regions can be found in (Turatsinze et al., 2008). For prediction of regions with a significant over-representation of sites (*i.e.*, CRMs, referred to as CRERS by this software), the CRER *p* value threshold was set at 0.0001 and CRER significance set at 3. When the same analyses were run on 150 random teleost fish sequences between 7000 and 20,000 bp in length, CRMs were found in less than 30% of cases. RSAT provides a graphical output of transcription factor binding sites that were further annotated and used in Fig. 1.

### Whole mount *in situ* hybridisation

Embryos were processed for whole-mount *in situ* hybridisation as previously described (Thisse et al., 1993).

### Morpholino injection

One-cell stage embryos were injected with, 0.5 pmol *tbx*16 (Bisgrove et al., 2005), 0.25 pmol ntla (Feldman and Stemple, 2001), 1 pmol tbx24 (Kawamura et al., 2008) or 0.25–1 pmol standard control morpholino (GeneTools).

### Expression con**s**tructs

The Ntla and Tbx16 coding regions were cloned in CS2+GR. Capped mRNA was synthesized from these CS2+GR constructs, pSP64TNtla (Schulte-Merker et al., 1992) and CS2+Tbx16 (Griffin et al., 1998).

### Protein synthesis inhibition and luciferase assays

One-cell stage embryos were injected with 150 pg of *ntla-GR* or *tbx*16*-GR* mRNA. Two hours prior to collection at 70–75% epiboly for *in situ* hybridization, embryos were treated with 10 μg/ml final concentration of cycloheximide for 30 min, then 20 μM dexamethasone for 1.5 h. For luciferase assays one-cell stage embryos were injected with 40 pg *deltaC*-CRM-luciferase construct (pGL3-promoter backbone (Promega) with wild type or mutated *dlc* CRM)*,* 0.75 pg of pCS2+Renilla and *ntla-GR* or *tbx*16*-GR* mRNA. Two hours prior to collection at 12 somite stage embryos were treated with 30 μg/ml cycloheximide for 30 min then with 60 μM dexamethasone for 1.5 h. 50 embryos for each condition were collected and assayed using the Dual Luciferase Assay (Promega) according to manufacturer's instructions. Each experiment was performed at least three times and data are reported as the mean fold change in luciferase activity compared to no mRNA (water) injection with standard error of the mean. The effectiveness of the cycloheximide treatment was tested by comparing levels of phosphorylated histone H3 (PH3, a marker of cell division) in treated and untreated embryos. Cycloheximide treatment resulted in down-regulation of anti-PH3 (NEB; 9701S) immunostaining compared to untreated embryos.

### Transient transgenesis

Thirty picograms of the *deltaC-*CRM-TK-37:mCherry::pXex:GFP construct (Garnett et al., 2009; wild type or mutated) or the empty TK-37:mCherry::pXex:GFP construct and 0.125 units I-SceI meganuclease were injected into the cytoplasm of early one-cell stage zebrafish.

### Immunostaining and cumulative expression diagrams

Twelve somite stage embryos which showed widespread GFP expression, indicating successful transgenesis, were fixed in 4% paraformaldehyde solution overnight then processed for mCherry protein expression by immunohistochemistry in batches of 80 (unless otherwise stated). Mouse anti-mCherry primary antibody (1:1000) and AP-coupled goat anti-mouse secondary (1:500) in TBS-SSDT solution (TBS, 1% BSA, 2% Goat serum, 1% DMSO and 0.1% Triton-X100) were used and NBT/BCIP used to develop the colour reaction. Sites of mCherry expression in the trunk and tail region were then recorded for each embryo and the percentage of embryos that were positive for mCherry expression in the *dlc* expression domain (tailbud, PSM, somites and notochord) was determined; these percentages are given in the figures. Cumulative data for each *dlc* CRM construct was then overlaid on a schematic zebrafish tail, giving an overall impression of the spatial pattern that each *dlc* CRM construct directs. Each injection experiment was carried out at least twice. Representative diagrams are shown.

### Fluorescent *in situ* and antibody staining

Protocols for double fluorescent *in situ*/*in situ* and *in situ*/immunostaining were as described (Cerda et al., 2009).

### Site directed mutagenesis

Mutations (from TCACACCT to GCAGGCCT) were induced in the T-box sites of reporter constructs using PCR (Table S1).

### Electrophoretic mobility shift assay (EMSA)

Radiolabelled probes (either wild type or mutated; see Table S1) were incubated with *in vitro* translated Ntla or Tbx16 protein (rabbit reticulocyte lysate; Promega) for 20 min at 4 °C in sucrose binding buffer (0.3 M sucrose, 20 mM Tris pH8, 5% Igepal, 62.5 mM NaCl, 4 mM MgCl_2_, 1 mM EDTA, 1× protease inhibitors). For competition reactions, the un-labelled competitor was pre-incubated with the protein for 20 min. For super-shifts, antibody was added after protein-probe incubation and incubated for a further 20 min. Reactions were run on a non-denaturing polyacrylamide gel and binding was visualized by autoradiography.

### GRN

Biotapestry (Longabaugh et al., 2005) was used to draw Fig. 6.

## Results

### Ntla and Tbx16 bind upstream of *dlc*

We previously performed ChIP-chip at mid-gastrula stages with anti-Ntla antibody that identified *dlc* as a potential target of this factor (Morley et al., 2009). These data show that Ntla binds a region −2.7 kb upstream of the *dlc* transcription start site (Fig. 1A). More recently ChIP-seq, using an antibody directed against Tbx16 which specifically recognizes Tbx16 in zebrafish embryos (Amacher et al., 2002; Garnett et al., 2009) also identified *dlc* as a potential target of Tbx16 (A. Nelson and F. Wardle, data not shown; Fig. 1A). ChIP-PCR at 75% epiboly using primers that bind within this region upstream of *dlc* (Fig. 1B, Table S1) confirmed that Tbx16 strongly binds upstream of *dlc* in the same region as Ntla (Fig. S1) and this led us to further investigate *dlc* regulation by these factors.

Inspection of the region bound by Ntla and Tbx16 reveals a cluster of T-box binding sites (TBS) including two that exactly match the known T-box 1/2 site consensus sequence: TCACACCT (Fig. 1B; referred to as cTBS1 and 2; (Kispert and Herrmann, 1993; Wilson and Conlon, 2002) and two that have a 1 bp or 2 bp mismatch (referred to as TBS3 and 4; Fig. 1B). Clusters of transcription factor binding sites are often associated with functional regulation and are termed cis-regulatory modules (CRMs). To test whether the clustering we observe is significant, and if a TBS-containing cluster is evolutionarily conserved in the vicinity of *dlc* orthologues in other species, we used the matrix-scan program from the RSAT suite of software, which detects transcription factor binding sites and predicts CRMs based on clustering of sites (Turatsinze et al., 2008). Using Ntla and Tbx16 position weight matrices (PWMs; Garnett et al., 2009; Morley et al., 2009) this analysis detects the four TBS upstream of zebrafish *dlc* and predicts the clustering to be significant, suggesting it is a CRM (pval<0.0001; Fig. 1C). Using this software, both the Ntla and Tbx16 PWMs identify cTBS1 and cTBS2 (purple bars) while the Ntla PWM identifies TBS3 (red bar) and Tbx16 PWM identifies TBS4 (blue bar). We then used this software to inspect the upstream region of *dlc* orthologues in four other teleost fish species (fugu, tetraodon, medaka and stickleback) and in two tetrapods (*Xenopus* and human). This found significant clustering of T-box binding sites (pval<0.0001) in fugu, tetraodon, stickleback, *Xenopus* and human (Fig. 1C), suggesting T-domain factor binding may be important in the regulation of *dlc* orthologues in these other species, although these sites are not within highly conserved non-coding elements (HCNEs; Frazer et al., 2004; data not shown). Medaka *dll3* upstream sequence contains several TBS, but they did not form clusters that reached the significance threshold (Fig. 1C), however such clusters of TBS can be found in the long introns of *dll3* (data not shown). These results show that Ntla and Tbx16 bind upstream of *dlc* in zebrafish during gastrulation and suggest that T-domain factors may also bind upstream of *dlc* orthologues in other species.

### Ntla and Tbx16, but not Tbx24, directly regulate *dlc* expression

**Fig. 2:**
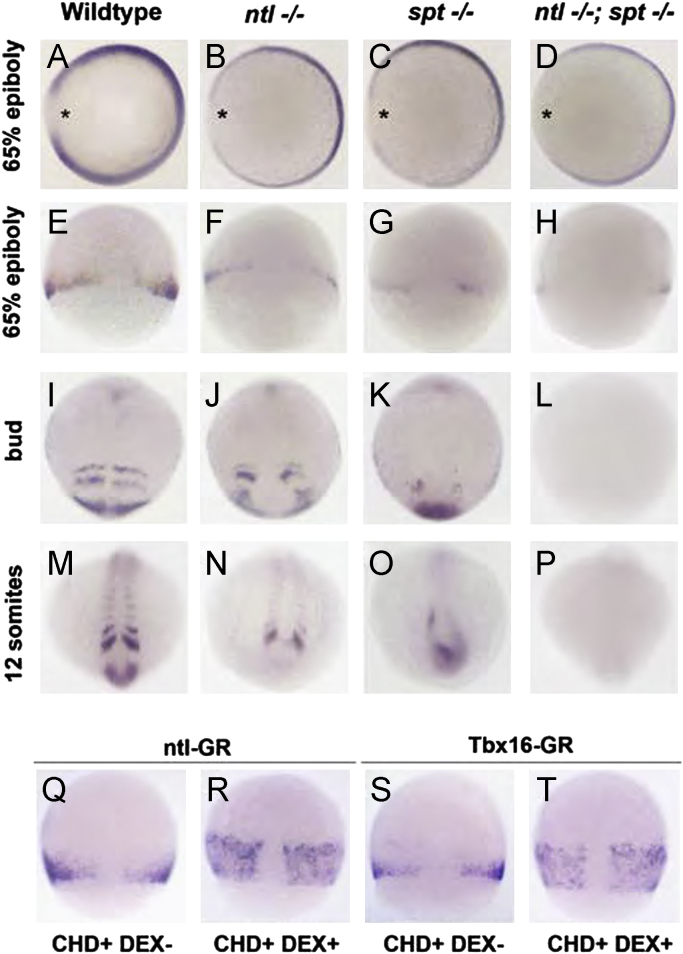
Ntla and Tbx16 directly regulate *dlc* expression (A)–(H) at 65% epiboly *ntla*^−/−^ and *spt*^−/−^ mutants have reduced dorsal-lateral expression of *dlc*, which is more pronounced in *ntla*^−/−^; *spt*^−/−^ double mutants (asterisks). The vegetal-animal width of the expression is also reduced in mutant embryos. (I)–(L) At bud stage, *dlc* expression in *ntla*^−/−^ mutants is similar to wild-type embryos, although somite shape is sometimes disrupted. Expression is much reduced in *spt*^−/−^ mutants and absent in *ntla*^−/−^; *spt*^−/−^ double mutants. (M)–(P) At 12 somite stage, *dlc* expression is absent from the posterior in *ntla*^−/−^ mutants, but remains in the somites. In *spt*^−/−^ mutants, expanded expression is seen in the tailbud, while expression is absent in *ntla*^−/−^; *spt*^−/−^ double mutants. (Q)–(T) Normal expression of *dlc* is seen in embryos injected with Ntla-GR or Tbx16-GR and treated with cycloheximide (CHD). Addition of dexamethasone (DEX) together with cycloheximide, results in expanded *dlc* expression at the margin, indicating *dlc* is directly regulated by Ntla and Tbx16. (A)–(D) show animal views, dorsal to the left; (E) - ((H) and (Q) - (T) show dorsal views, animal to the top. (I) - (P) show dorsal views, anterior to the top.

We next asked if Ntla and Tbx16 regulate expression of *dlc* in zebrafish embryos by analysing its expression in mutant embryos (Fig. 2 and Table S2). In wild type embryos at 65% epiboly, *dlc* expression is seen in the ventral-lateral margin but is excluded from the dorsal margin. In *ntla*^−/−^ and *spt*^−/−^ single mutant embryos expression is down-regulated on the dorsal side (Fig. 2A–C, E–G), and is further down-regulated in *ntla*^−/−^; *spt*^−/−^ double mutants (Fig. 2D, H). The width of the marginal expression in the vegetal-animal direction is also reduced in mutant embryos at this stage even though a normal germ ring forms (Halpern et al., 1993; Kimmel et al., 1989 and data not shown). At bud stage wild type *dlc* expression is seen in the tailbud and in stripes in the anterior paraxial mesoderm (Fig. 2I). In bud stage *ntla*^−/−^ mutant embryos, *dlc* is expressed in a pattern similar to wild type embryos, although the shape of the forming somites is often disrupted (Fig. 2J). In *spt*^−/−^ mutant embryos, *dlc* expression is seen in the tailbud and in a few cells adjacent to the midline which may correspond to the clusters of cells observed in the paraxial region of the trunk in these embryos (Fig. 2K; Kimmel et al., 1989). Expression is absent from *ntla*^−/−^; *spt*^−/−^ double mutants in the trunk and in the tailbud (Fig. 2L). At later somitogenesis stages (12ss), *dlc* expression in *ntla*^−/−^ mutant embryos is absent from the very posterior of the embryo, consistent with the lack of a normal tailbud at this stage in the mutant, but expression remains in the somites. In *spt*^−/−^ mutants, expression is observed in the characteristic expanded tailbud region, while expression is absent from double mutants (Fig. 2M–P), consistent with a lack of tissue in the posterior of the embryo in these mutants. These observations were confirmed in embryos morphant for *ntla* and/or *tbx*16 (Fig. S2). These results show that *dlc* is a target of Ntla and Tbx16 in the germ ring during gastrulation stages. In contrast, *dlc* expression remains in the somites (*ntla*^−/−^ mutants) or tailbud (*sp*t^−/−^ mutants) suggesting neither factor is required individually for expression of *dlc* at later stages, and since *ntla*^−/−^; *spt*^−/−^ double mutants lack the mesodermal tissues *dlc* is expressed in, it is not possible to make any conclusions about whether they regulate expression together. However, since these factors work redundantly to form trunk and tail somites (Amacher et al., 2002) it is possible that they also work redundantly in regulating gene expression.

Tbx24 (mutated in *fused somites*; *fss*) is another T-domain factor which is expressed in the lateral margin during gastrulation and in paraxial mesoderm during somitogenesis, and which is required for segmentation (Nikaido et al., 2002). Since Tbx24 has been implicated in the regulation of another cyclically expressed gene, *her1* (Brend and Holley, 2009), we tested whether *dlc* expression is altered in *tbx24* morphant embryos. We found that expression of *dlc* was not down regulated in *tbx*24 morphants during gastrulation and somite stages (Fig. S2), suggesting that Tbx24 does not regulate *dlc* expression, although we saw compacted expression of *dlc* in the somites of these embryos consistent with the *tbx*24 morphant phenotype.

Since the results above suggested that Ntla and Tbx16 may play a role in regulating *dlc* expression at gastrula stages, we next determined if they could directly regulate *dlc* expression using injection of dexamethasone-inducible GR-fusion constructs (Ntla-GR or Tbx16-GR) and protein synthesis inhibition with cycloheximide (Kolm and Sive, 1995; Martin and Kimelman, 2008). We found that, when activated by dexamethasone, both Ntla-GR and Tbx16-GR are able to expand the expression of *dlc* in the margin during gastrulation (data not shown) and this was also the case in the presence of cycloheximide (Fig. 2R and T), indicating that both Ntla and Tbx16 directly regulate *dlc* expression. However, we did not see ectopic expression in the animal pole region, which may suggest additional factors are required for activation but are limited to the margin, alternatively, other factors may repress expression in the animal pole.

### A cis-regulatory region upstream of *dlc* drives reporter expression in PSM and somites

**Fig. 3:**
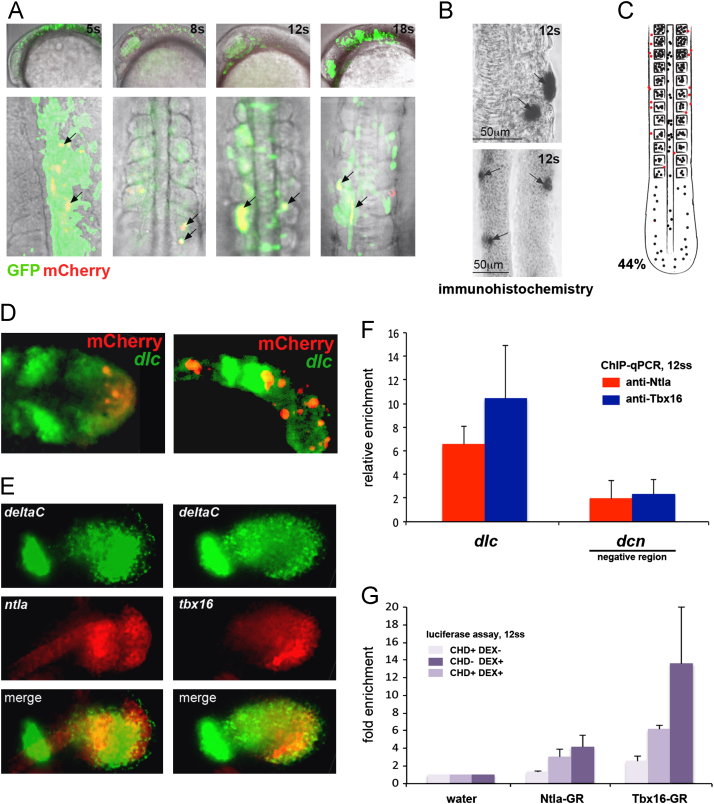
*dlc* CRM drives expression of reporter in tailbud, PSM and somites. (A) Confocal images of transient transgenics at 5, 8, 12 and 18 somite stages (5s, 8s, 12s, 18s) show mCherry expression (red; under the control of the *dlc* CRM) overlaps with GFP (green; under the control of a ubiquitous promoter) in the somites (arrows). Conversely, although strong GFP expression is seen in the nervous system, including the eye, mCherry expression is not seen here. (B) mCherry protein is detected in the somites by immunohistochemistry in 12 somite stage embryos (arrows). (C) Sites of mCherry expression detected by immunohistochemistry at 12ss (examples in (B)) were recorded and used to produce a representative composite of mCherry expression from 80 embryos. Black dots represent mCherry-expressing cells in the somites, PSM, tailbud or notochord; red dots represent mCherry expressing cells outside these tissues. 44% of embryos which expressed GFP also expressed mCherry in the somites, PSM, tailbud and/or notochord at 12ss. (D) Co-localisation of mCherry protein driven by the *dlc* CRM in the talibud (red) and endogenous *dlc* mRNA at 12 somite stage (green) shows overlapping expression. Left image is a dorsal view, right image is a lateral view. (E) Co-localisation of *dlc* mRNA (green) with *ntla* or *tbx16* mRNA (red) at 12 somite stage shows overlapping expression in the tailbud. Lateral views are shown. (F) ChIP-PCR on 12-somite stage embryos using primers that amplify a region within the *dlc* CRM (see Fig. 1A and Table S1) show that *dlc* is bound by Ntla (red bars) and Tbx16 (blue bars) at this stage. For comparison, enrichment of binding around a negative region 2.2 kb upstream of *dcn* (see Table S1 for primer sequence) is shown. (G) Ntla-GR and Tbx16-GR activate luciferase expression through the *dlc* CRM in the presence of dexamethasone and cycloheximide at 12ss, indicating regulation by these factors is direct.

In order to test whether the CRM we identified upstream of *dlc* is able to drive expression in embryos we cloned a 493 bp region encompassing the TBS cluster (Fig. 1B) into a mCherry reporter vector that contains a minimal promoter (TK-37; Garnett et al., 2009). We then injected this reporter into one-cell stage embryos to create transient transgenic embryos, and assessed mCherry protein expression by fluorescent microscopy and immunohistochemistry (Fig. 3A, B and D). The reporter also contains GFP under the control of a ubiquitous promoter enabling us to monitor successful transgenesis (Garnett et al., 2009). As a control for any baseline activation, we injected the vector and found that it drives mosaic expression of GFP during gastrulation and somitogenesis stages, but does not drive expression of mCherry (80/80 embryos with GFP expression and no mCherry expression; data not shown). Upon injection of the *dlc* CRM reporter construct we observed mosaic GFP expression during gastrulation stages, indicating transgenesis was successful, but we did not observe mCherry expression either by fluorescence or immunostaining at this stage. Instead, expression of mCherry is first observed at 3–4 somite stage (ss) in the tailbud, PSM and somites. Expression of mCherry continues in the tailbud, PSM and somites throughout somitogenesis and is seen until at least until 24 h post fertilization (Fig. 3A and data not shown). We also observed some expression in the notochord, another site of *dlc* expression during somitogenesis (Smithers et al., 2000). In addition to mesodermal expression, *dlc* also initiates in the nervous system during somite stages and is particularly strongly expressed in the neural retina from around 13ss (Smithers et al., 2000), however we do not see mCherry expression in the central nervous system including the retina at later somite stages (Fig. 3A). Occasionally, expression of mCherry was observed in some cells overlying the somites/PSM trunk; these may correspond to the scattered cells seen in ventral lateral ectoderm in wild-type embryos (Smithers et al., 2000) or may be ectopic spots of expression. Because this analysis was performed in transient transgenic embryos, we attempted to produce a more complete understanding of reporter gene expression by recording the expression of mCherry at the 12 somite stage across multiple embryos to produce a composite picture of expression as previously described (Muller et al., 1997; Woolfe et al., 2005). For this assay we selected embryos at 12ss, which were broadly expressing GFP across the whole embryo, including in the somites, and fixed these for immunohistochemistry with an anti-mCherry antibody. Examples of clones of cells expressing mCherry, as detected by this method, are shown in Fig. 3B. These sites of mCherry expression were then counted and their position within the somites, PSM, tailbud, notochord, or other region in the posterior of the embryo recorded for each embryo, then superimposed on a schematic diagram of the posterior of a 12ss embryo (see materials and methods for further explanation). An example of this is shown in Fig. 3C, where 44% of embryos (35/80) expressed mCherry in regions of endogenous *dlc* expression (another example is shown in Fig. 5A where 41% of embryos (33/80) expressed mCherry in regions of endogenous *dlc* expression). The composite picture shows expression of mCherry in the tailbud, PSM and somites and notochord (Fig. 3C), consistent with what we saw in individual embryos. However, since this expression is not seen in all transgenic embryos (*i.e.*, those expressing GFP) this suggests that the activity of the 493 bp CRM is influenced by the surrounding genomic landscape it finds itself in, and is not a strong enhancer that can work in any position.

We next assessed if the reporter expression, as assayed by mCherry protein, co-localized with endogenous *dlc* expression during somitogenesis. Fig. 3D shows overlap of *dlc* mRNA expression with mCherry protein in the tailbud, PSM and somites. The expression of the reporter is thus consistent with the known expression of *dlc* at somite stages.

This CRM drives expression only during somitogenesis in wild type embryos but our initial ChIP analysis was carried out at gastrulation stages. Therefore we asked if Ntla and Tbx16 are in a position to regulate the CRM during somite stages *in vivo* and if so whether this regulation is direct. First, we investigated whether Ntla and Tbx16 are present at the same time and place as *dlc* using fluorescence double *in situ* hybridization, and found expression of both *ntla* and *tbx16* colocalizes with that of *dlc* in the tailbud region of the 12ss embryo (Fig. 3E). We next tested binding of Tbx16 and Ntla to the CRM at 12ss by ChIP-qPCR using anti-Ntla and anti-Tbx16 antibodies (Amacher et al., 2002; Morley et al., 2009; Schulte-Merker et al., 1992). The results show that binding is enriched at the *dlc* CRM at this stage compared to a control region (Fig. 3F), albeit that the enrichment is lower than at gastrula stages. This is likely to be because fewer cells as a total of the whole embryo express Ntla and Tbx16 at 12ss compared to gastrula stages, resulting in less DNA being immunoprecipitated and leading to a weaker signal. Thus both Ntla and Tbx16 are appropriately placed in the embryo and on the genomic DNA to regulate *dlc* expression in the tailbud during somitogenesis.

Finally, we asked if the *dlc* CRM directly responds to Ntla and Tbx16 during somitogenesis. We co-injected the *dlc* CRM luciferase reporter with Ntla-GR and Tbx16-GR. Ntla-GR and Tbx16-GR were then activated by treatment with dexamethasone in the presence or absence of cycloheximide for two hours prior to collection at 12ss. We find that Ntla-GR weakly activates luciferase expression 3–4 fold in the presence of dexamethasone with or without cycloheximide, suggesting Ntla is able to directly bind and activate the *dlc*-CRM at 12ss (Fig. 3G). Tbx16-GR also activates luciferase expression approximately 6-fold in the presence of dexamethasone, but interestingly this activation is increased when cycloheximide is added (Fig. 3G), which may suggest that Tbx16 normally activates a repressor which can inhibit transcription from this CRM. Further investigation will be required to confirm and identify any such potential regulator.

Together these results show that this CRM is sufficient to drive expression of the reporter in the tailbud, PSM, somites and notochord during somitogenesis, a pattern consistent with the expression of *dlc*, and that Ntla and Tbx16 bind and directly activate its expression during somitogenesis. Since this CRM does not drive expression during gastrulation, despite Ntla and Tbx16 binding the CRM and directly activating *dlc* expression at this time, other regulatory regions must be implicated in the initiation of *dlc* expression in the ventro-lateral mesoderm during gastrulation.

### Tbx16 and Ntla are required together to drive reporter gene expression *in vivo*

**Fig. 4:**
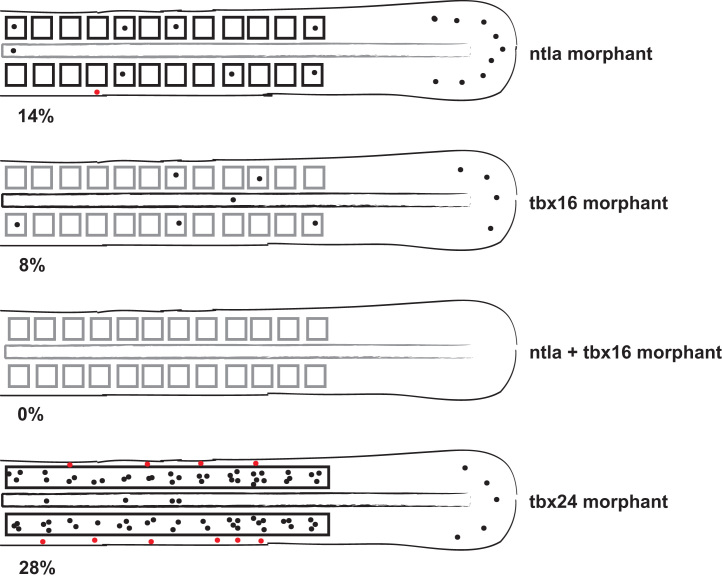
Tbx16 and Ntla are required together to drive reporter gene expression *in vivo*. Expression of mCherry protein after injection of the *dlc* CRM mCherry reporter. mCherry is down regulated in *ntla* or *tbx16* single morphant embryos compared to injection into wild type embryos at 12ss, while expression of mCherry in *ntla*+*tbx16* double morphant embryos is abolished (80 embryos were scored for each condition). Notochord is not present in *ntla* morphants (indicated in grey); anterior trunk somites are not present in *tbx16* morphants (indicated in grey). Expression of mCherry protein after injection of the *dlc* CRM mCherry reporter in *tbx24* morphant embryos (60 embryos) is mildly down-regulated, although somite boundaries are indistinct in *tbx24* morphant embryos (indicated by open box). Black dots represent mCherry-expressing cells in the tailbud, PSM, somites or notochord; red dots represent mCherry-expressing cells outside these tissues. Percentages indicate how many embryos expressed mCherry in the tailbud, PSM, somites and/or notochord.

The previous sections show that Ntla and Tbx16 bind the *dlc* CRM *in vivo*, so if the CRM is regulated by these two factors *in vivo* we would expect reporter activity to be down-regulated during somite stages in *ntla* and/or *tbx16* morphant embryos. To test this we injected the mCherry reporter into one-cell embryos together with *ntla* and/or *tbx16* morpholinos. Fig. 4 shows that reporter expression is reduced compared to wild type embryos in the tailbud and somites in single morphants, while expression is completely lost in *ntla*/*tbx16* double morphants. These results are consistent with the endogenous expression of *dlc* in *ntla*, *tbx16* and *ntla*/*tbx16* morphant embryos during somitogenesis, although since trunk and tail mesodermal tissue is lost in the double morphants we cannot conclude to what extent these factors are required together for CRM expression. We also asked if expression of the reporter was affected by knockdown of *tbx24* and found that mCherry protein was expressed in the somitic mesoderm of *tbx24* morphants, albeit in a smaller number of embryos than in wild type embryos, suggesting that the *dlc* CRM does not strongly respond to Tbx24 regulation *in vivo* (Fig. 4). These results suggest that both Tbx16 and Ntla regulate activation of the *dlc* CRM during somitogenesis.

### T-box sites are required for binding and reporter gene activation

**Fig. 5:**
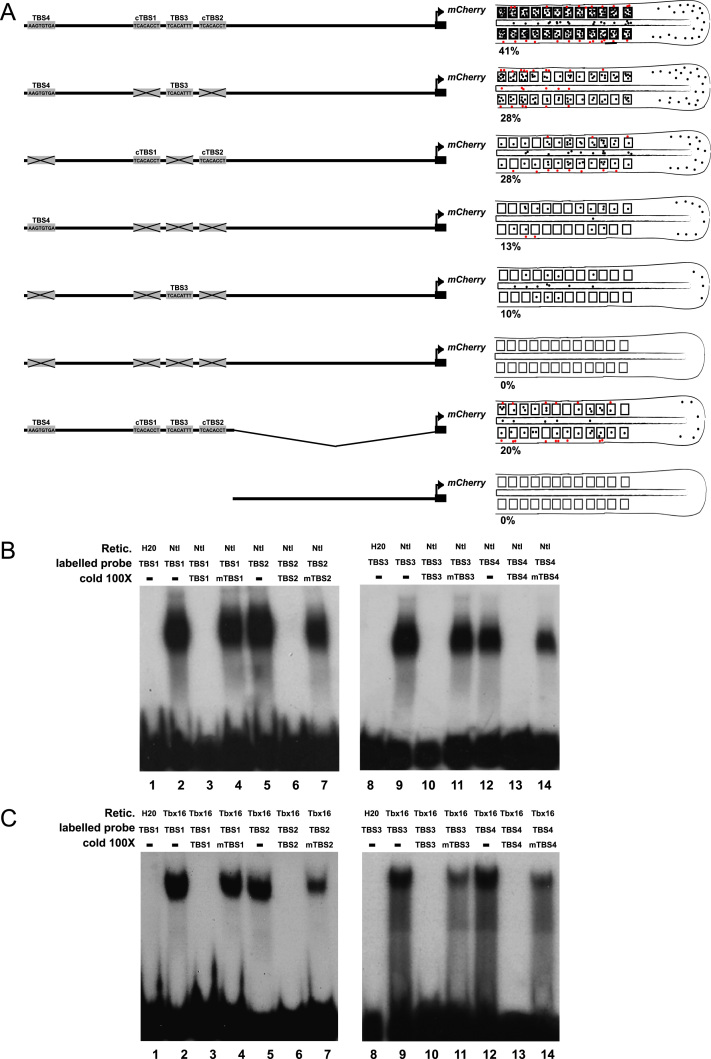
T-box sites are required for *dlc* CRM expression *in vivo*. (A) Expression of mCherry protein after injection of wild type or mutated *dlc* CRM mCherry reporters as shown on the left. The wild type construct drives expression in 41% of cases, while mutating the consensus TBS (cTBS1 and cTBS2) decreases but does not abolish mCherry expression. Mutating TBS3 and 4 also decreases but does not abolish expression of mCherry. Mutating combinations of three different TBS further decreases expression, but only when all four sites are mutated is mCherry expression abolished completely. The most distal half of the *dlc* CRM is able to drive expression of mCherry in the tailbud and somites, although at a lower level than the complete region, while the proximal half does not drive expression of mCherry. Black dots represent mCherry-expressing cells in the tailbud, PSM, somites or notochord; red dots represent mCherry expressing cells outside these tissues. Percentages indicate how many embryos expressed mCherry in the tailbud, PSM, somites and/or notochord out of a total of 80 embryos. (B) EMSA showing Ntla binding to wild type cTBS1 (lane 2), cTBS2 (lane 5), TBS3 (lane 9) and TBS4 (lane 12). Sequences of probes can be found in Fig. 1B and Table S1. This binding is competed by cold wild type probes (lanes 3, 6, 10, 13) but not by corresponding mutated probes (lanes 4, 7, 11, 14). (C) The same pattern of binding is seen with Tbx16 and wild type and mutant probes.

Next we asked if the T-box binding sites in the *dlc* CRM are required for expression of the reporter using our transient transgenic assay at 12ss. Mutations in the consensus TBS (cTBS1 and 2) resulted in decreased expression of the mCherry reporter but did not abolish expression *in vivo* (Fig. 5A). We reasoned that the other T-box binding sites (TBS4 and 4) in the cluster may act redundantly, and therefore made mutations in these sites. Fig. 5A shows that mutation of all four T-box binding sites in the cluster leads to complete loss of reporter gene expression in transient transgenic animals. Finally, we cut the reporter region in half, leaving one region containing all T-box sites and another region without. The half CRM containing the TBS cluster is able to weakly drive mCherry expression, while the other half does not drive expression, suggesting that the proximal CRM, which does not contain any T-box binding sites, enhances expression activation but is not able to drive expression alone. These results suggest that all four sites are needed for robust CRM activation, but that there is some functional redundancy between sites since a reporter with only TBS3 and TBS4 intact is able to drive expression of mCherry to similar levels as one with only cTBS1 and cTBS2 intact, and a single site is still able to drive some mCherry expression in the mesoderm.

Since all four TBS were required for *dlc* CRM activation at 12ss in the transient transgenic assay, we next tested binding of Ntla and Tbx16 using electrophoretic mobility shift assays (EMSA). We confirmed that Ntla and Tbx16 bind to all four 30 bp probes containing an individual T-box binding site (Fig. 5B and C lanes 2, 5, 9, 12) and that this binding is specific, since the corresponding unlabelled probes are able to compete for binding with labelled probe (Fig. 5B and C lanes 3, 6, 10, 13). However, the corresponding mutated unlabelled probes do not compete (Fig. 5B and C lanes 4, 7, 11, 14). In addition, mutation of each site abolishes binding of Ntla or Tbx16 to labeled mutated probe (data not shown). These results indicate that both Ntla and Tbx16 are able to bind cTBS1, cTBS2, TBS3 and TBS4 individually. We also assayed binding to a longer 72 bp radiolabelled probe that contains the two consensus T-box binding sites and TBS3. In this assay, both Ntla and Tbx16 are able to bind the wild type probe and this binding is competed by unlabeled probe (Fig. S3A and B lanes 3, 4). Unlabelled probe that is mutated for one of the consensus TBS (leaving the other cTBS and TBS3 intact) competes for binding with labeled wild type probe (Fig. S3A and B lanes 5, 6), suggesting Ntla or Tbx16 continue to bind to the remaining sites. However, when both consensus sites (cTBS1 and 2) are mutated, leaving TBS3 intact, the unlabelled probe is unable to compete for Ntla or Tbx16 binding with labeled probe (Fig. S3A and B, lane 7). This suggests that although Ntla and Tbx16 are able to bind TBS3 when on its own (Fig. 5A and B), in the context of the larger probe this interaction is not strong enough to produce a shift. Finally, since Ntla and Tbx16 are together required for CRM activation, we investigated whether they form a complex by co-incubating Ntla and Tbx16 protein with the 72 bp probe. However, these proteins do not appear to form a complex together, instead forming separate complexes that can be super-shifted independently (Fig. S3C and D).

Taken together, these results show that Ntla and Tbx16 are able to bind all the T-box binding sites in the CRM upstream of *dlc*, and together activate expression from this region, although there appears to be functional redundancy between sites.

## Discussion

We have shown here that Ntla and Tbx16 directly regulate the expression of *dlc* during gastrulation and somite stages. All three factors are expressed in the margin at gastrulation and in the tailbud during somite stages (Smithers et al., 2000; Fig. 3E) and both Ntla and Tbx16 bind a CRM upstream of *dlc* at these stages. However, the CRM we have isolated does not drive expression during gastrulation, which suggests that other regulatory regions are required for expression at this stage. Instead, the CRM we have isolated drives expression of a reporter in the tailbud, PSM and somites of the developing embryo during somitogenesis and its activity requires a cluster of T-box binding sites. These findings implicate Ntla and Tbx16 in a direct role in segmentation through regulating *dlc* expression.

Despite binding of Ntla and Tbx16 to the *dlc* CRM at gastrulation stages, and direct activation of *dlc* by these factors, the *dlc* CRM does not drive reporter gene expression during gastrulation. This may be because other regions that bind Ntla and Tbx16 are involved in early activation of *dlc*. Intriguingly, further inspection of the ChIP-seq data shows two other peaks of Tbx16 binding at 22 kb and 28 kb downstream of the *dlc* gene (data not shown), although further investigation will be required to discover if these play any role. Alternatively, the *dlc* CRM may be involved in early expression together with other transcription factors that bind outside the CRM. In this scenario, activation of endogenous *dlc* during gastrulation is seen (Fig. 2R and T) but early activation cannot be recapitulated when assaying the role of the CRM on its own since the other sites are not present.

The *dlc* CRM drives reporter gene expression in a pattern consistent with endogenous *dlc* expression during somite stages, *i.e.*, in the tailbud, PSM, somites and notochord. A caveat to this is that endogenous *dlc* mRNA is normally expressed in the posterior of the mature somite, however, we see expression of mCherry protein throughout the somites. This may be due to the perdurance of the mCherry protein, which has a long-half life in zebrafish (∼24 h), thus the mCherry that is detected may be protein that was made in the tailbud/PSM at an earlier time point, rather than reflecting *de novo* transcription of mCherry in the somites. Unfortunately, attempts at generating a stable line in which to test reporter mRNA expression have so far been unsuccessful, due to apparent silencing of the construct. Without this we are unable to test whether the CRM can drive mRNA expression in the posterior somites and in a cyclic manner in the posterior PSM in the same way as endogenous *dlc*. Cyclic expression depends on repression as well as activation, and our experiments suggest that Tbx16 may activate a repressor of the *dlc* CRM during somitogenesis. In the PSM, repression of cyclic genes is generally mediated by Hairy/E(Spl)-related proteins (Giudicelli et al., 2007), and it may be that Tbx16 is able to activate one or more members of this family which act indirectly or directly on the CRM; further investigation is required to confirm this. Interestingly, the expression of tbx16 itself is cyclic (Krol et al., 2011) which could directly lead to cyclic expression of target genes, such as *dlc*. We note also that this CRM is downstream of the *timm50* gene (Fig. 1A), whose expression becomes restricted to the somites during somitogenesis, so it is possible that this CRM regulates expression of *timm*50 rather than, or as well, as *dlc*. However expression analysis by microarray and *in situ* hybridization suggest that *timm*50 expression is not regulated by Ntla and/or Tbx16, since its expression is not down regulated in mutant embryos when *dlc* expression is (Garnett et al., 2009; data not shown).

Consistent with our observations that Ntla or Tbx16 alone do not regulate *dlc* expression during somite stages, since expression remains in the trunk somites in *ntla*^−/−^ mutants and in the tailbud in *spt*^−/−^ mutants, we also do not see complete loss of *dlc* CRM transgene expression in individual *tbx*16 or *ntla* morphants at the 12 somite stage. This is also consistent with the known redundant role of Ntla and Tbx16 in trunk and tail somite formation (Amacher et al., 2002; Goering et al., 2003). However the observation that Ntla and Tbx16 appear to form separate complexes on the DNA (Fig. S3; see also Garnett et al., 2009), may suggest that Ntla and Tbx16 work independently in regulating downstream targets during normal embryogenesis. It may be that Ntla and Tbx16 act in the same cells but bind individually to the CRM to regulate gene expression. Alternatively, Ntla may bind the CRM in a different cell population to Tbx16 in the posterior of the embryo. Unfortunately, due to the nature of this whole embryo ChIP protocol, we are unable to differentiate binding in separate tissues. Tbx24, which is able to bind T-box sites (Brend and Holley, 2009), may also play some role in regulating this CRM during somite stages since there was a decrease in mCherry expression in Tbx24 morphant embryos, although it does not appear to play as large a part as Ntla and Tbx16.

We found a differing requirement for Ntla, Tbx16 and the four TBS depending on the assay we employed. In transient transgenics, all four sites appear to be required for expression and can partially functionally substitute for each other. For instance, if cTBS1, cTBS2 and TBS3 are mutated, TBS4 alone is able to drive low levels of mCherry expression. Correspondingly, Ntla and Tbx16 are able to specifically bind all four TBS individually in an EMSA. However, in an EMSA using a larger probe containing cTBS1, cTBS2 and TBS3, TBS3 was not able to bind Ntla or Tbx16 when the other sites were mutated, suggesting the affinity for TBS3 is not as strong as the other sites in this assay. By the ChIP assay *in vivo* the T-box binding sites are too close to distinguish whether one site is bound rather than another, and while the transgenic mCherry reporter assay may be the closest to a wild type situation, with the result that all four sites functionally substitute for each other, it does not provide clues as to which sites are normally preferred by Ntla and Tbx16 *in vivo*. Nevertheless, our results show Ntla and Tbx16 regulate *dlc* expression during early zebrafish development, and point to a role for the cluster of T-box binding sites in this regulation.

**Fig. 6:**
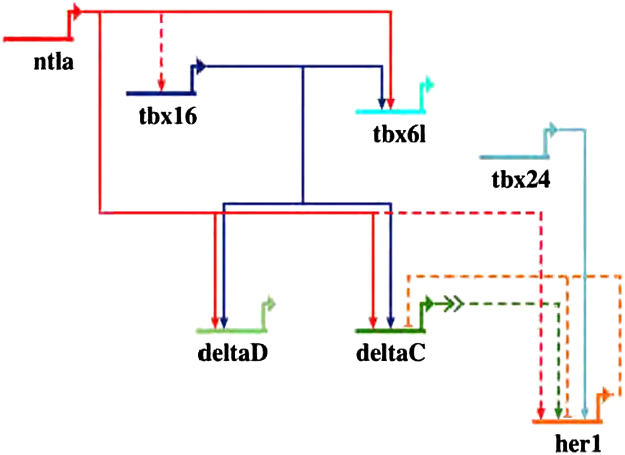
A gene regulatory network connects T-box factor activity with Notch signaling. Dashed lines indicate that a genetic interaction has been seen between two factors; solid lines indicate additional assays have shown direct regulation between two factors. Ntla binds upstream of *dlc*, *dld, tbx*6l, *tbx*16 and *her*1, and directly regulates expression of *dlc, dld, and tbx6l.* Tbx16 binds upstream and directly regulates *dlc, dld* and *tbx6l*. Tbx24 directly regulates *her1*. A regulatory loop exists between *dlc* and *her1*.

Other studies have implicated Ntla and Tbx16 in patterning the somites through regulating other genes required for segmentation (Amacher et al., 2002; Garnett et al., 2009). For instance, Ntla and Tbx16 regulate *her1* and *dld* expression (Amacher et al., 2002; Garnett et al., 2009). Ntla and Tbx16 bind upstream of d*ld*, and several TBS in this upstream region are required for expression in the posterior of the embryo, whilst *her*1 expression is down regulated in *ntla*^−/−^; *spt*^−/−^ mutants. *her*1 expression has also been shown to be dependent on a TBS in the region −2.9 kb to −3.3 kb upstream of the *her*1 transcription start site, and Tbx24 is implicated in this regulation (Brend and Holley, 2009). However, the role of other T-domain factors was not assessed in this study, leaving room for the possibility that other T-domain factors also regulate *her1* expression directly. In support of this, we see Ntla binding, although not Tbx16 binding, in the −2.9 kb to −3.3 kb upstream of *her1* (Morley et al., 2009). The Notch pathway and its targets thus appear to form a network with T-box genes in the regulation of somite formation, which we have represented as a network diagram in Fig. 6.

In mouse, the T-domain factor Tbx6 is expressed early in development in the primitive streak during gastrulation (Chapman et al., 1996), whilst also regulating somite patterning and segmentation later in development. Mutations in Tbx6 cause loss of paraxial mesoderm, while Tbx6, in conjunction with Wnt or Notch signaling, regulates expression of genes involved in somite patterning and segmentation including *Dll1*, *Msgn1* and *Mesp2* (Chapman and Papaioannou, 1998; White et al., 2003; Wittler et al., 2007; Yasuhiko et al., 2006; Yasuhiko et al., 2008). Our results support the idea that in zebrafish a combination of T-box factors, including zebrafish Ntla and Tbx16, may have the same activity as the single Tbx6 factor in mouse and may cooperate to regulate segmentation gene targets (Goering et al., 2003; Wardle and Papaioannou, 2008).

## Author contributions

LJ and FCW performed ChIP experiments, ACN performed ChIP-seq analysis. LJ performed all other experiments and wrote the paper. FCW designed the study and wrote the paper.

## Figures and Tables

**Figure:**
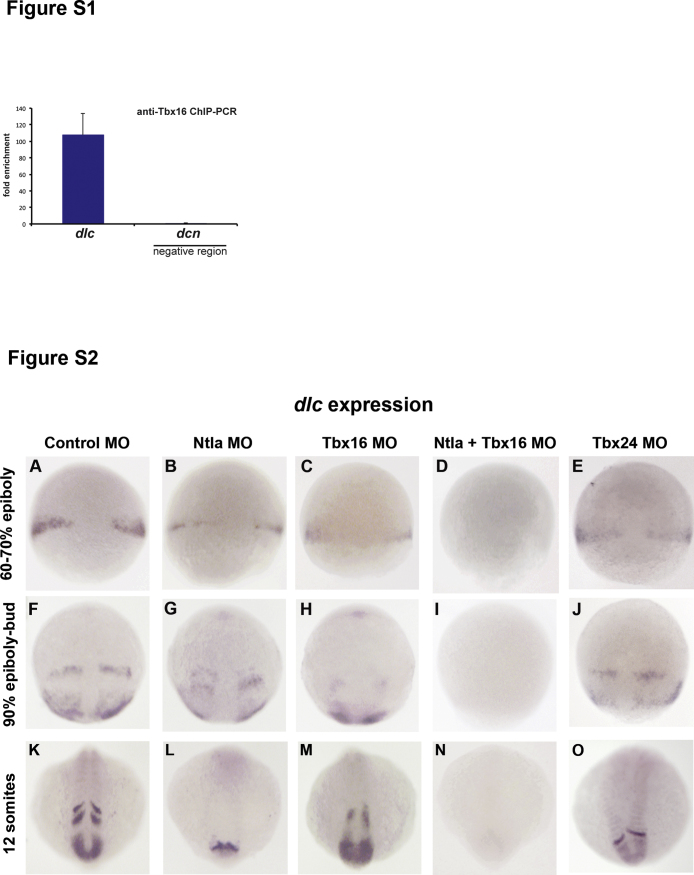


**Figure:**
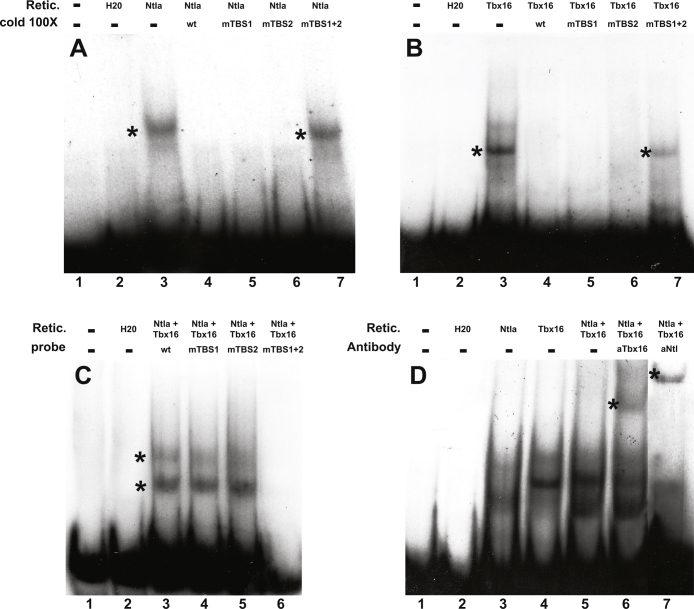


## Footnotes

Appendix A Supplementary data associated with this article can be found in the online version at doi:10.1016/j.ydbio.2012.07.002.
