# Supplementary material for: A cis-regulatory module upstream of deltaC regulated by Ntla and Tbx16 drives expression in the tailbud, presomitic mesoderm and somites
Source: Dev Biol. 2012 Nov 1;371(1-10):110–20. doi: 10.1016/j.ydbio.2012.07.002 (PMC3460241; doi:10.1016/j.ydbio.2012.07.002)
Supplement: Supplementary file 2 — Supplementary material [file mmc2.docx]

**Table S2**: *dlc* *in situ* hybridization patterns

| stage | **65% epiboly** | | **bud** | | **12 somites** | |
| --- | --- | --- | --- | --- | --- | --- |
| cross | wildtype | abnormal | wildtype | abnormal | wildtype | abnormal |
| *ntla*^+/-^ | 31 (69%) | 14 (31%) | 66 (73%) | 18 (27%) | 35 (73%) | 13 (27%) |
| *spt*^+/-^ | 31 (78%) | 9 (22%) | 27 (70%) | 12 (30%) | 32 (76%) | 10 (23.8%) |
| *ntla*^+/-^*/spt*^+/-^ * | 14 (61%) | 9 (39%) | 27 (51%) | 25 (49%) | 43 (61%) | 27 (39%) |

*ratios (wt: *ntla*^-/-^: *spt*^-/-^ : *ntla*^-/-^; *spt*^-/-^):

65%: 14:7:2 (n.b. at 65% the *ntla*^-/-^ phenotype was indistinguishable from the spt^-/-^ phenotype)

bud: 27:10:11:4

12s: 43:11:12:4

| stage | **65% epiboly** | | **bud** | | **12 somites** | |
| --- | --- | --- | --- | --- | --- | --- |
| morpholino | wildtype | abnormal | wildtype | abnormal | wildtype | abnormal |
| *ntla* | 2 (5%) | 40 (95%) | 9 (21%) | 33 (79%) | 10 (12%) | 74 (88%) |
| *tbx16* | 5 (13%) | 34 (87%) | 7 (17%) | 35 (83%) | 0 (0%) | 37 (100%) |
| *ntla*+ *tbx16* | 0 (0%) | 40 (100%) | 0 (0%) | 39 (100%) | 0 (0%) | 64 (100%) |
| *tbx24* | 41 (100%) | 0 (0%) | 43 (100%) | 0 (0%) | 0 (0%) | 35 (100%) |
| control | 24 (100%) | 0 (0%) | 30 (100%) | 0 (0%) | 23 (100%) | 0 (0%) |
